# Rate, not selectivity, determines neuronal population coding accuracy in auditory cortex

**DOI:** 10.1371/journal.pbio.2002459

**Published:** 2017-11-01

**Authors:** Wensheng Sun, Dennis L. Barbour

**Affiliations:** Department of Biomedical Engineering, Washington University in St. Louis, St. Louis, Missouri, United States of America; McGill University, Canada

## Abstract

The notion that neurons with higher selectivity carry more information about external sensory inputs is widely accepted in neuroscience. High-selectivity neurons respond to a narrow range of sensory inputs, and thus would be considered highly informative by rejecting a large proportion of possible inputs. In auditory cortex, neuronal responses are less selective immediately after the onset of a sound and then become highly selective in the following sustained response epoch. These 2 temporal response epochs have thus been interpreted to encode first the presence and then the content of a sound input. Contrary to predictions from that prevailing theory, however, we found that the neural population conveys similar information about sound input across the 2 epochs in spite of the neuronal selectivity differences. The amount of information encoded turns out to be almost completely dependent upon the total number of population spikes in the read-out window for this system. Moreover, inhomogeneous Poisson spiking behavior is sufficient to account for this property. These results imply a novel principle of sensory encoding that is potentially shared widely among multiple sensory systems.

## Author summary

Neurons act together to encode information such as the nature of a sensory stimulus. The number of neurons used for individual stimuli and the nature of the encoding used are not well understood. Higher sensory areas have been observed to respond sparsely to sensory stimuli, meaning that only a relatively small number of neurons fire action potentials (or “spikes”) when any particular stimulus is present. Sparse spiking activity is present in primary auditory cortex but only after a sound has been playing for some period of time. Dense spiking, however, is present at stimulus onset. We found that each action potential in primary auditory cortex contributed approximately the same amount of information about a tone stimulus, which resulted in more accurate encoding from the dense onset spiking. The later sparse spiking retained stimulus information but required a longer time to read it out. This arrangement allows sensory stimuli to be identified rapidly yet represented efficiently for extended periods, while neurons still retain sensitivity to novel stimuli. Dense and sparse coding may therefore work together dynamically in order to represent complex, temporally overlapping sensory content.

## Introduction

Our current understanding of sensory information processing in the brain has been built heavily upon the observations of neurons that respond only to particular sensory inputs. One classical and common conception is that neurons with higher selectivity, i.e., responding to a narrower range of sensory input parameters, provide more information about sensory input [1–4]. In auditory cortex, selectivity of single neurons increases as spiking evolves following the onset of a stationary sound stimulus. Specifically, during the first tens to hundreds of milliseconds following stimulus onset, neurons tend to respond to a large range of sounds. Average spiking rates are high during this transient epoch and settle to lower sustained values during the later epoch. Differential adaptation leads to many fewer neurons active during the sustained response epoch, resulting in a sparser representation and, correspondingly, a neuron population of higher selectivity [5–8]. This feature of neuronal adaptation following stimulus onset is shared among multiple sensory systems [9–11]. Based on this selectivity difference, the current theory regarding neuronal responses in auditory cortex posits that the relatively unselective (i.e., dense) onset responses predominately encode the presence of sounds whereas the more selective (i.e., sparse) sustained responses predominately encode the identity of sounds [5, 12, 13].

The approach of interpreting informativeness based on neuronal response selectivity, although intuitively appealing, contains potential caveats upon closer consideration. For example, due to the limited size of individual neuronal receptive fields, stimulus information must be encoded collectively by multiple neurons [14, 15]. Therefore, a larger population of highly selective neurons would be required to encode an entire stimulus space compared to less selective neurons.

Current theory predicts that the sustained response epoch of auditory cortical responses should carry more information on average than the onset response epoch because stimulus identification is a less constrained task than detection. Here, we tested this prediction by investigating the sound encoding dynamics of a population of 171 neurons in marmoset primary auditory cortex (A1). Our results contradicted current theory and implied a novel sensory encoding principle potentially applicable to other sensory systems.

## Results

### Onset response epoch contains more stimulus information than sustained response epoch in short windows of the same length

**Fig 1:**
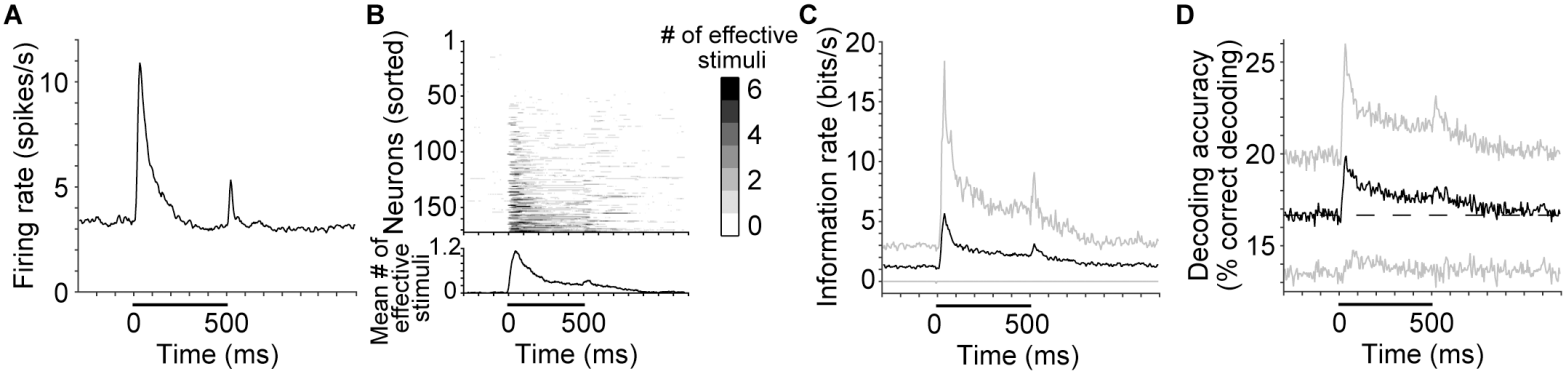
Response pattern and information content of single A1 neurons. (A) Mean spiking rate across all neurons (*N* = 171 neurons) and stimuli (6 tones). Spiking rates were calculated in 15-ms time windows. Abscissa values mark window centers. Horizontal black bar denotes sound duration. These conventions are followed throughout all relevant figures unless otherwise stated. (B) (Upper plot) The number of stimuli eliciting significant increases in spiking rate (i.e., effective stimuli) for each neuron; neurons sorted by the average number of effective stimuli. (Lower plot) The mean number of effective stimuli across the neural population. Note that the number of effective stimuli is inversely related to neural response selectivity. (C) Rate of mutual information between stimuli and neural responses of single neurons over time, calculated in 15-ms time windows. Black curve shows the mean value across the neural population (*N* = 171 neurons); gray curves show the 10% and 90% quantiles. (D) Percentage of correctly decoded trials by single neurons. Black curve shows the mean accuracy across the neural population; gray curves show 1 standard deviation above and below the mean (*N* = 171 neurons). Dashed line represents chance performance in this and all other classification result plots. Numerical data and analytical results can be found at osf.io/xhmus/. A1, primary auditory cortex.

To provide an equal stimulus condition for comparing different response epochs, we tested each neuron’s responses with a fixed set of stationary, pure-tone stimuli. The stimulus set consisted of six 500-ms pure tones at 85 dB sound pressure level (SPL) spaced 1 octave apart in frequency. Linear ramps of 5-ms duration were applied to the beginning and end of each sound to alleviate the effect of spectral splatter at these transitions. We began by confirming that our data set contains the same response characteristics observed in previous studies. Indeed, we found that mean stimulus-induced spiking rate (Fig 1A) was highest at stimulus onset while neural response selectivity was lowest (Fig 1B), confirming previous observations [5, 8, 16, 17]. The low mean spiking rate during the sustained response epoch resulted from a combination of activated and suppressed neural responses relative to spontaneous neural activity.

To evaluate the amount of stimulus information about the tonal frequency within the neural responses, we first quantified the mutual information between the stimulus set and short segments of spiking responses from each neuron, comparing the results over time relative to sound onset. Note that the nonzero mutual information value during silence before sound onset represents the residual bias and variance in the mutual information calculation after the correction procedure [18–20] and serves as a gross baseline to gauge the true information rate values in other epochs. Contrary to prediction, average neural responses during the onset response epoch are far more informative than responses during the sustained response epoch (Fig 1C).

Because any particular decoder does not necessarily make use of all the information available in a signal [21], we next sought to construct a single-neuron decoder of stimulus identity to test the theory’s assertions directly (see Materials and methods). A decoder based on template matching via Euclidean distance was constructed in this case [22]. Consistent with the measurement of mutual information, the accuracy of single-neuron decoding during the onset response epoch was much better than during the sustained response epoch, even though absolute decoding accuracy was relatively poor in both epochs with this decoder (Fig 1D).

**Fig 2:**
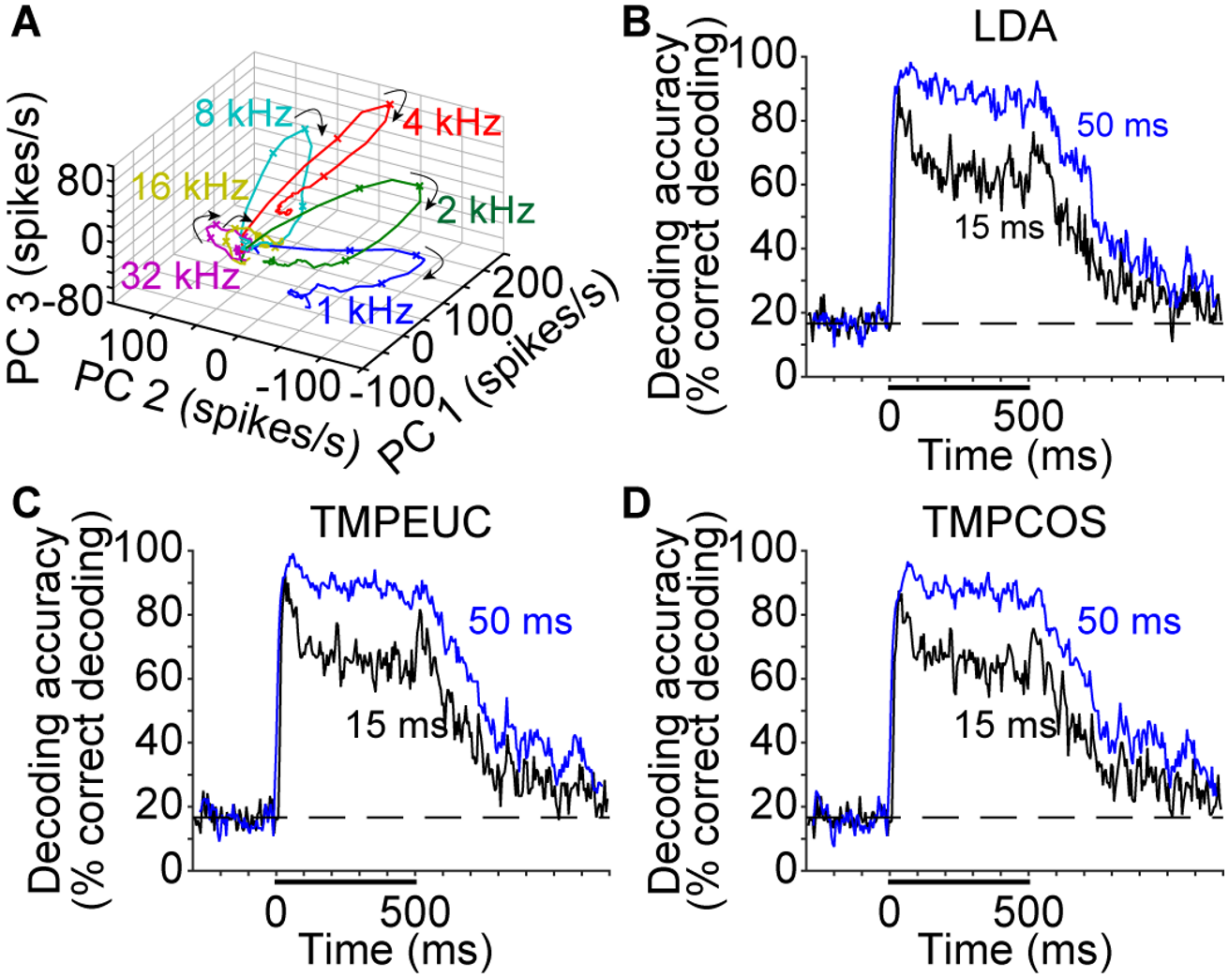
Response pattern and decoding performance of neuronal population. (A) Population neural response trajectories obtained by projecting the mean spiking rate response across trials onto the first 3 principal components of neural activity. Trajectories are color-coded by sound identity. Neural activities from 30 ms before sound onset to sound offset are plotted. Crosses mark the time points of 30, 50, and 100 ms after sound onset. Arrows denote the direction of trajectory evolution. Visually, trajectories are distinguishable both by the absolute distances between them and by the relative angles between the hyperplanes in which they are embedded. (B-D) Accuracy of decoding sound identity from single trial responses of the neuron population over time. Each panel shows results of decoding with a different method: (B) LDA, (C) TMPEUC, and (D) TMPCOS. Black and blue curves show the results of decoding using 15-ms and 50-ms read-out windows, respectively. Numerical data and analytical results can be found at osf.io/xhmus/. LDA, linear discriminant analysis; PC, principal component; TMPCOS, template matching with angular distance metric; TMPEUC, template matching with Euclidean distance metric.

Actual brain function is constructed from the collective activity of a population of neurons. Depending on the amount of information overlap among neurons, the information content of a neural population is not necessarily equal to the simple summation of individual neurons’ information. To fully characterize the neural response dynamics, we next pooled the neurons across experiments and examined them collectively as a neural population [11, 23–25]. Fig 2A shows the mean population spiking behavior over time projected onto the first 3 principal components of neural population activity [11, 23, 24, 26]. Individual stimulus responses are visually distinguishable in both the onset and sustained response epochs.

In order to quantify this result, we again constructed classifiers to predict stimulus identity over time, this time from responses of the neural population. Unlike single-neuron decoding, more methods are available for decoding neural population responses. To ensure that the results reflect the inherent information content of the neural population instead of the properties of a particular type of decoder, 3 distinct types of decoding strategies were evaluated. These 3 strategies were linear discriminant analysis (LDA) [27, 28], template matching based on a Euclidean distance metric (TMPEUC) [11, 23, 25, 29], and template matching based on an angular distance metric (TMPCOS) [29, 30]. The outputs of these classifiers built independently for neural responses in short time windows can be seen in Fig 2B–2D. In every case, classification performance was best at onset. This outcome reinforces our analysis of spiking information in Fig 1C and 1D and further contradicts the theory that stimulus identification is mainly coded by sustained spiking. Note that compared to the large difference in mean single neuron information content and decoding accuracies between the 2 epochs in Fig 1C and 1D, the disparity of the population decoding accuracies between the 2 epochs in Fig 2B–2D (black curves) is considerably smaller. Population decoders therefore achieve greatly improved stimulus classification performance by considering information jointly encoded among neurons.

### The effects of window length on decoding accuracy

Having established that short segments of neural responses in both onset and sustained response epochs contain stimulus identity information, the question arises whether these neural responses can be temporally combined to improve decoding accuracy. As a first step toward clarifying this potential, we tested the decoding accuracy of the population with response segments in a longer read-out window (Fig 2B–2D blue curves). Indeed, decoding accuracy improved for all 3 decoders, with greater improvement for the sustained response epoch, presumably because accuracy in the onset response epoch already approached saturation in shorter windows. Note that as a comparison, decoding accuracy during the silence period before stimulus onset did not increase under the longer window length. Decoding accuracy for multiple window lengths can be compared directly in S1 Fig.

**Fig 3:**
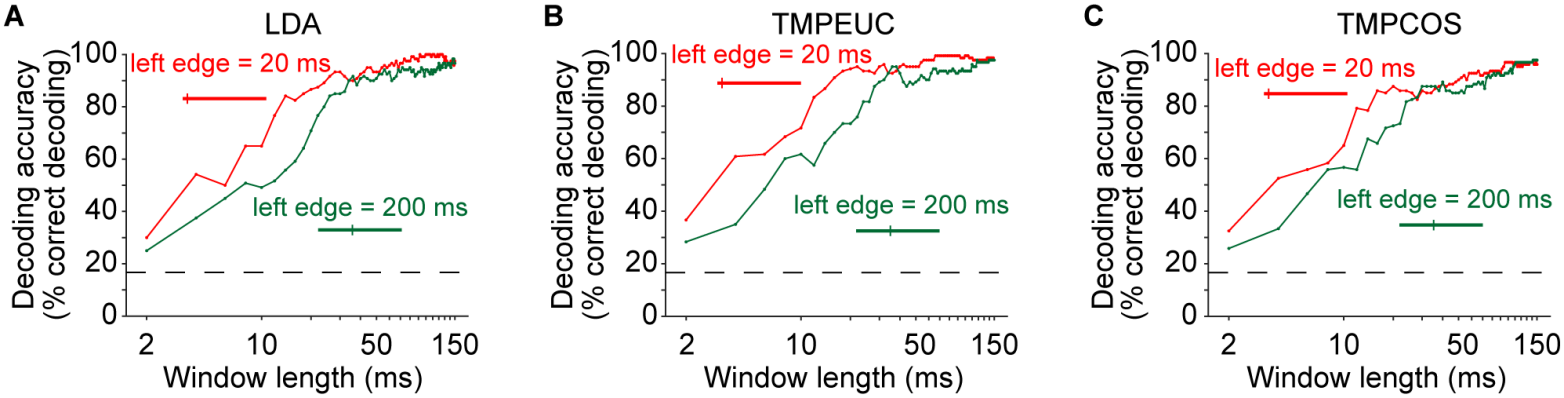
Decoding accuracy under different window lengths for 2 example time points. Windows in red and green curves start at 20 ms (onset response epoch) and 200 ms (sustained response epoch) after sound onset, respectively, and extend rightward to achieve the indicated length. Each panel shows results of a different decoding method: (A) LDA, (B) TMPEUC, and (C) TMPCOS. Numerical data and analytical results can be found at osf.io/xhmus/. LDA, linear discriminant analysis; TMPCOS, template matching with angular distance metric; TMPEUC, template matching with Euclidean distance metric.

To obtain a more complete picture of the stimulus information encoded throughout the 2 epochs, we next evaluated decoding accuracy at a time point within each epoch under a range of read-out window lengths (Fig 3A–3C; S2 Fig). Two observations become immediately apparent. First, for both onset and sustained time points, decoding accuracy generally increased with window length, until reaching comparable saturation performance. Second, for every window length leading to unsaturated performance, the decoding accuracy of the onset response epoch was greater than that of the sustained response epoch. Thus, responses within the 2 epochs contain a similar amount of information about the tonal frequency of the stimuli, but to read out this information, a much shorter time window is sufficient for the onset response epoch.

**Fig 4:**
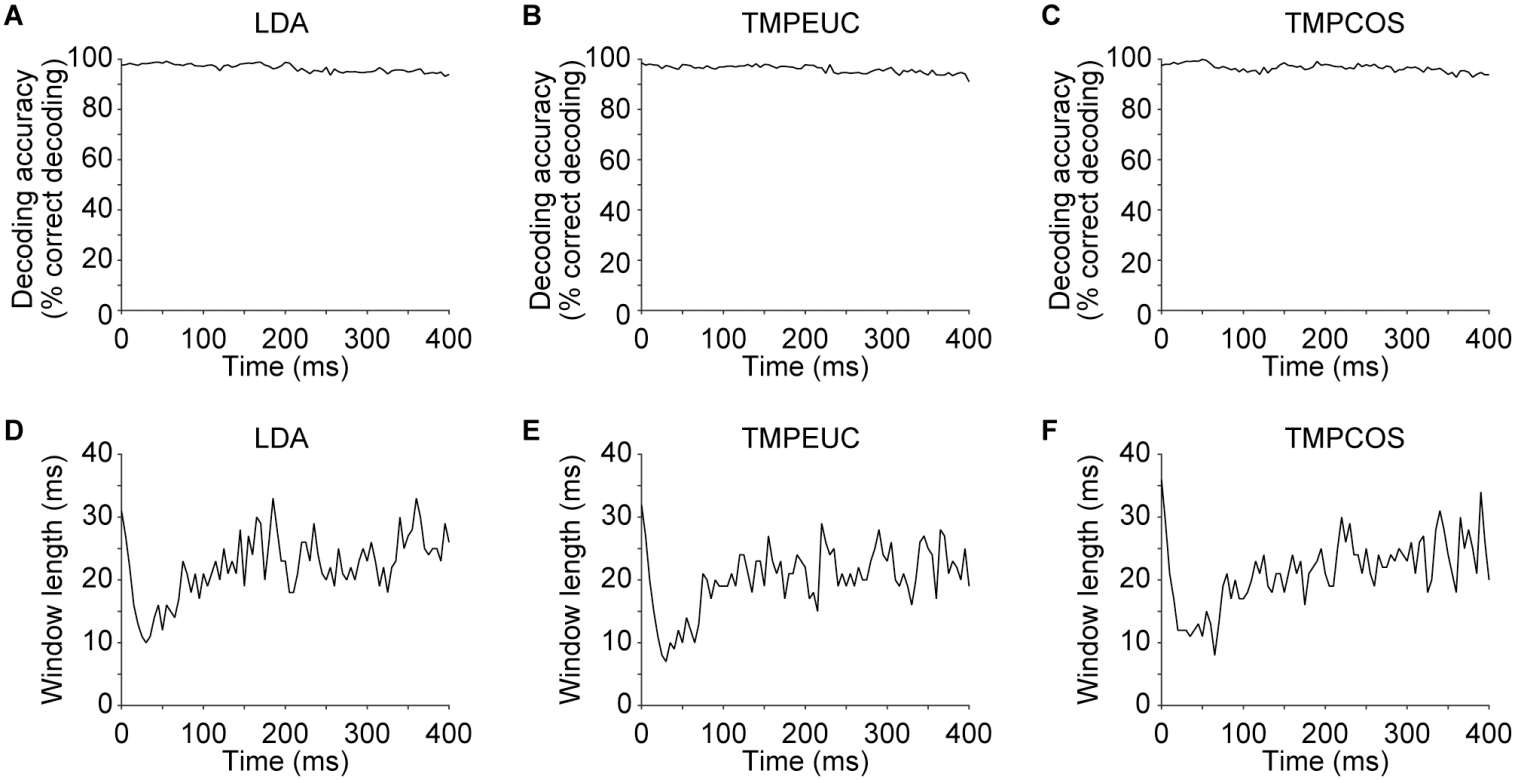
Effects of window lengths at different time points. (A-C) Saturation decoding accuracy for windows starting at different time points from 0 to 400 ms after sound onset in 5-ms increments for the 3 types of decoders. (D-F) Minimum window length at which decoding accuracy was equal to or above 80% of the saturation decoding accuracy for the 3 types of decoders. Numerical data and analytical results can be found at osf.io/xhmus/. LDA, linear discriminant analysis; TMPCOS, template matching with angular distance metric; TMPEUC, template matching with Euclidean distance metric.

We further verified that saturation decoding accuracy was similar for all decoders and across the entire stimulus duration (Fig 4A–4C) and determined that the minimum window length required for decoding accuracy to achieve 80% of its saturation level was smallest during the onset response epoch (Fig 4D–4F). This result extends the observations at the 2 individual time points in Fig 3A–3C. Note that the minimum window lengths required for windows beginning within the first 15 ms after sound onset had higher values because these windows contained an initial time period when stimulus-induced spiking activity had not yet begun due to neural response latency.

### The constant spike count encoding phenomenon

**Fig 5:**
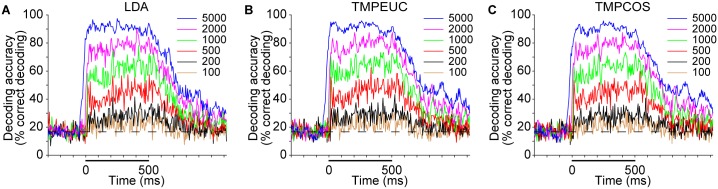
Constant decoding accuracy using a fixed number of spikes. Decoding accuracy in analysis windows containing a fixed number of spikes across all neurons and stimuli. Each curve shows results of decoding with a different spike count number. Decoding accuracy is plotted against the beginning time of each read-out window. Each panel shows results of a different decoding method: (A) LDA, (B) TMPEUC, and (C) TMPCOS. Numerical data and analytical results can be found at osf.io/xhmus/. LDA, linear discriminant analysis; TMPCOS, template matching with angular distance metric; TMPEUC, template matching with Euclidean distance metric.

With these observations of stimulus encoding performance in auditory cortex, new questions arise about the nature of dynamic stimulus encoding in sensory systems generally. The similar information content yet lower information rate of the sustained response epoch, for example, raises the intriguing possibility that sensory stimuli may be encoded in such a way that decoding accuracy is primarily dependent simply upon the total number of action potential spikes distributed across a population of tuned sensory neurons. For convenience of reference, we refer to this as “the constant spike count hypothesis.” To test this new hypothesis, we first constructed read-out windows by accumulating into them predetermined numbers of spikes and then compared decoding accuracies over time using all 3 decoders (Fig 5A–5C). We found the decoding accuracy at a given spike count to be essentially equivalent over time during sound presentation for all 3 decoders. Furthermore, the decoding accuracy systematically improved when more spikes were included. Therefore, a sensory neuron population can achieve an arbitrary degree of decoding accuracy over a stimulus set simply by accumulating a particular number of stimulus-encoding spikes into a classifier. Because each of the 3 different classifiers we evaluated decodes information in a very different way, the consistency of this result across decoding strategy implies a robust population code whereby accuracy is relatively insensitive to the form of the decoder, as long as a sufficient number of encoding spikes is included.

### Poisson spiking statistics support constant spike count encoding

What features of a sensory system give rise to universal decoding given similar per-spike information? Template matching, for example, assigns a response to the stimulus eliciting the most similar mean responses, whereas LDA relies upon linearly projecting the data onto a few dimensions that can maximally separate out responses to different stimuli. We suspected that the nature of the variability of individual neuronal spiking output could contribute to the similar decoding accuracy we observed. One way to quantify a neuron’s spiking variability is to calculate the variance of its spiking rate estimate in a given window over repeated recordings. The same amount of variance however, could have different effects on neural encoding depending on the magnitude of the spiking rate itself. A better metric would be the coefficient of variation (CV), which normalizes the standard deviation of the spiking rate with the mean spiking rate and reflects the ratio of “noise” relative to the magnitude of the “signal.” Larger CV values represent less reliable spiking activity.

For Poisson spiking neurons, it can be shown that the CV has a simple relationship with the neuron’s true spiking rate and the window length of observing its spiking activity. CV is simply the inverse of the square root of the product of the 2 terms (see S1 Text). Note that the product of the true spiking rate and the observation window length gives the expected spike count in that window. Therefore, the reliability of Poisson spiking in any given observation window is determined simply by the expected number of spikes in that window. To state this property another way, a high-spiking Poisson neuron can convey its true rate with a particular accuracy in a shorter time interval than would be required by another Poisson neuron with a lower true rate. A population of adaptive (i.e., inhomogeneous) Poisson-spiking sensory neurons would therefore be able to encode sensory stimuli at stimulus onset with the smallest window length when average spiking rates are highest, just like our observations in auditory cortex.

**Fig 6:**
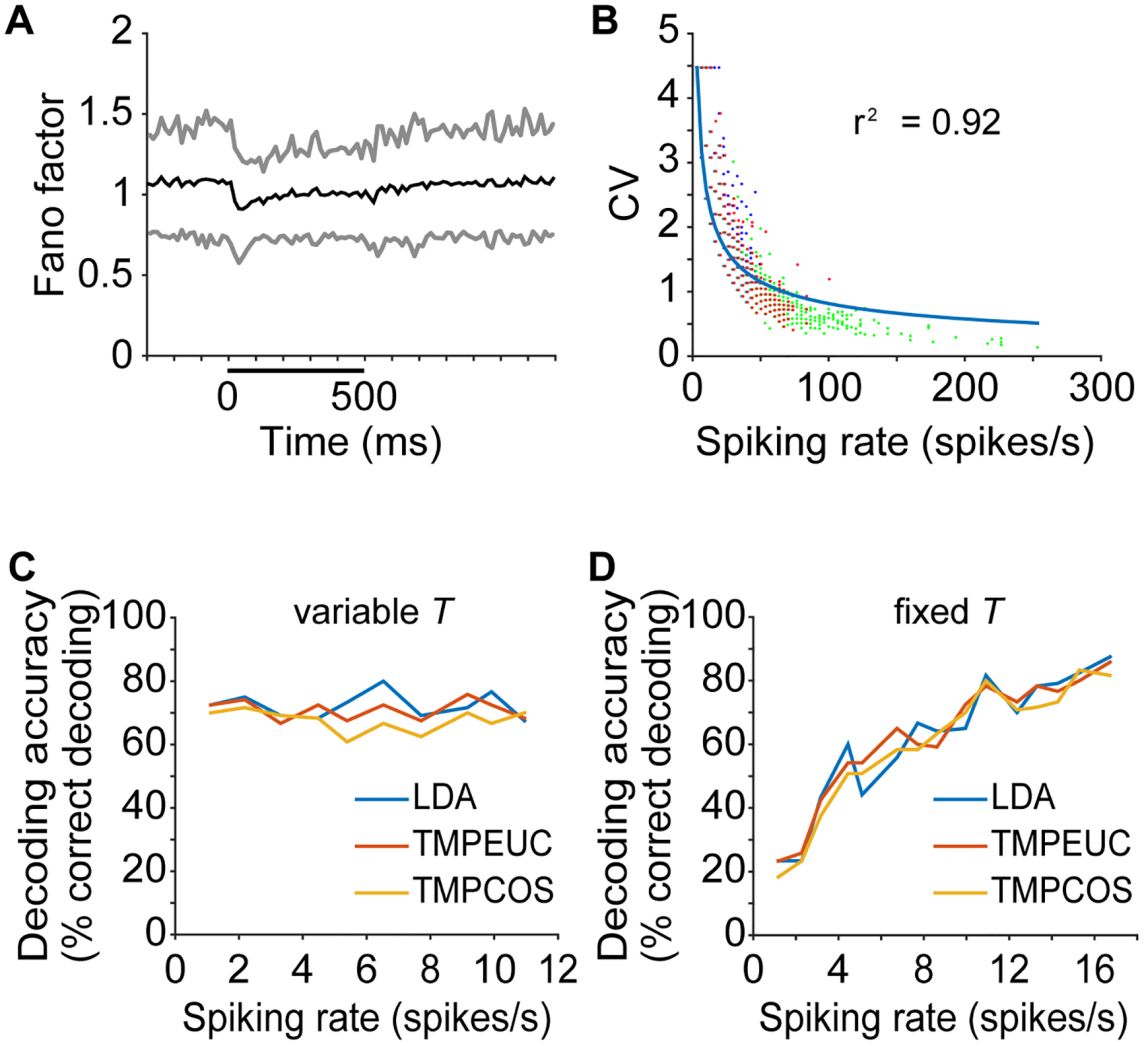
Poisson spiking model supports constant spike count hypothesis. (A) Mean Fano factor of the neural population in 15 ms windows is shown in black. Gray curves show the value of 1 standard deviation above and below the mean. (B) Neuronal CV plotted against the mean spiking rate for all recorded neurons in 15-ms windows. Blue, green, and red dots are each from neural responses within the ranges of [−300, 0) ms, [0, 200) ms, and [200, 500] ms relative to sound onset time, respectively. To better display overlapping data points, blue and red dots are displaced 0.5 spikes/s to the left and right, respectively, of their original positions. The solid line shows the relationship between these 2 measures for ideal Poisson neurons. (C) Sound decoding accuracy of a simulated Poisson-spiking neural population with mean spiking rate uniformly adapted to different values. The read-out window length (*T*) was adjusted to maintain the same expected spike count in all conditions. Lines of different colors show results of different decoding methods. (D) Sound decoding accuracy of simulated Poisson-spiking neural populations with different mean spiking rate values in a fixed read-out window length (*T*). The same plotting notation of panel C is followed. Numerical data and analytical results can be found at osf.io/xhmus/. CV, coefficient of variation; LDA, linear discriminant analysis; TMPCOS, template matching with angular distance metric; TMPEUC, template matching with Euclidean distance metric.

To verify that this property of Poisson-spiking neurons could help explain our observed relationship between decoding accuracy and spike count, we sought to confirm that our data are indeed Poisson-like. We calculated the Fano factor (the ratio of spike count variance to mean) over time for each neuron in the population. The mean Fano factor is near unity for silence, onset and sustained epochs, confirming population Poisson-like behavior over time (Fig 6A). A likelihood ratio test for the null hypothesis of Poisson distribution [31] revealed that none of the recorded neural responses deviated significantly from Poisson distribution (*P* > 0.05 for all neurons after multiple comparisons correction). We next examined the relationship between the CV and the mean spiking rate for our population of neurons and found that the distribution reasonably matches what would be expected for a collection of Poisson spiking neurons (Fig 6B), with some balanced deviation from Poisson at the highest and lowest spiking rates. Therefore, the average behavior of our neural population is indeed Poisson-like.

One question about a Poisson-spiking population is whether adaptive sparsification is necessary for neurons to convey information in the adapted epoch when rates drop to spontaneous values. Could uniform rate adaptation also bring about similar decoding accuracy for a given sensory neuron population? We addressed this question first by theoretically analyzing how an inhomogeneous Poisson-spiking neuron’s discriminability between 2 stimuli changes with uniform rate adaptation. It can be shown that the discriminability of a Poisson-spiking neuron does not change after its spiking rates elicited by the 2 stimuli are adapted down by the same ratio, as long as the observation window is lengthened accordingly (see S2 Text). Therefore, uniformly adaptive Poisson spiking neurons appear to be sufficient to account for the constant spike count hypothesis.

To confirm this analysis, we simulated the effects of uniform adaptation on the encoding ability of a purely Poisson-spiking neuron population (Fig 6C). Consistent with the theoretical reasoning above, when the observation window length is adjusted to maintain the same spike count over the simulated population, uniform rate adaptation does not change the population decoding accuracy. These results further suggest both that inhomogeneous Poisson spiking is sufficient to account for the constant spike count hypothesis and that the sparsification resulting from differential adaptation is not necessary for this encoding ability. Sparsification under these conditions therefore probably provides its classically described advantages [32, 33].

### Analysis with subset data and additional data further supports the conclusions

The stimulus set used to demonstrate more accurate encoding per unit time during the onset response epoch consisted of tones with frequencies spaced wider than the animal’s behavioral discrimination threshold. This stimulus set leaves open the question of whether tones with frequency differences closer to behavioral thresholds may be encoded more accurately during the sustained response epoch. This possibility exists because in single neurons the relative differences in spiking induced by 2 very similar stimuli may be much smaller during onset response than sustained response (i.e., differential selectivity). Current theory would predict in such a case that sustained spiking would provide more accurate decoding.

To evaluate our new corollary, we extended our analysis in 2 ways. First, we created a condition similar to narrowly spaced tone delivery by constraining the analysis described above only to neurons from the existing data set with the broadest tuning. These 16 neurons all responded to 1 or more tones during the sustained response epoch and a larger number of tones, 2 or more, during the onset response epoch. This scenario mimics the situation in which neurons with narrower tuning are driven by tones spaced in frequency closer to behavioral threshold. With this subset, we again tested the decoding accuracy with windows containing a given number of spikes. As with the full data set, responses from this subset of neurons having differential tone selectivity over time again showed the same decoding accuracy over time in windows containing the same spike count (S3 Fig). In fact, differential selectivity in this case seems to be an irrelevant factor for the validity of the constant spike count hypothesis because similar results held even when examining the rest of the neurons excluding the 16 most differentially selective neurons (S4 Fig).

The analysis above does not compare the maximum informational capacity of the 2 epochs, however. The constant spike count hypothesis predicts that given a larger number of neurons yielding more population spikes per unit time, both onset and sustained responses in such cases should be able to identify tones with more narrowly spaced frequencies. Our second additional analysis was, therefore, to evaluate frequency decoding performance among tones of finer frequency differences with a larger population of neurons aggregated from previous experiments. Two data sets were evaluated for this purpose. The first contained 478 neurons tested with 9 tones spaced 1/8 octave apart between 8 to 16 kHz. The second was a subset of 376 neurons tested with 17 tones spaced 1/16 octave apart between 8 to 16 kHz. Tones delivered in both data sets were 100 ms long, which allowed evaluation of the onset responses but not the sustained responses. S5 Fig shows the results, in which onset responses exhibited high or intermediate decoding accuracy, not the low decoding accuracy that current theory predicts. Furthermore, decoding accuracy began to fall off near the end of the stimulus interval, just as it occurred near 100 ms after sound onset with the main data set in Fig 2, which is consistent with lower sustained decoding accuracy. S6 Fig plots the normalized decoding accuracies for the 3 different frequency spacing to demonstrate similar decoding performance. The ideal experiment would require sampling a larger number of neurons stimulated by tones of longer duration to provide a direct comparison between the 2 response epochs. This experiment remains an interesting topic for future investigation.

## Discussion

We studied the population encoding properties of marmoset A1 neurons in response to a tonal stimulus set. We found that an equivalent amount of information about the tonal frequency of the stimuli was contained in intervals having the same spike count across both the onset and sustained response epochs. The Poisson nature of spike trains helps to explain this constant spike count encoding property. Higher information transfer rates could support more rapid information processing, while lower information transfer rates could reduce energy requirements through a lower spiking rate. Our constant spike count hypothesis postulates that simultaneous system constraints of (1) the fundamental biophysical properties of how spikes are generated, manifested as Poisson-like spiking, (2) the behavioral need for rapid stimulus identification at onset and less rapid readout as a stimulus continues unchanged, and (3) the metabolic need for efficient stimulus encoding throughout the remainder of the stimulus interval, have collectively given rise through evolutionary processes to the spiking rate dynamics we observe. An advantage of this strategy is that it is simultaneously both fast and efficient.

A less obvious advantage of such a sensory system is that encoding accuracy could be modulated for any decoder by altering the total number of spikes produced across the population for any stimulus. Fig 6D demonstrates that when simulating purely Poisson-spiking neurons responding to our 6 stimuli at a fixed observation window length, higher average discharge rates lead to higher decoding accuracy. A sensory system with excess rate capacity (by having many normally low-spiking neurons, for example) could therefore speed up decoding simply by adjusting all the neurons in the population to higher discharge rates, or possibly by recruiting more neurons to generate more spikes. The latter is exactly what happens during stimulus transitions, giving rise to strong onset responses.

Given that we have firmly established accurate A1 encoding of tone frequency during the onset response epoch given a sufficient number of spikes, a logical question would be why sustained responses exist at all. The continuing representation of an ongoing stimulus might be necessary or at least convenient from a decoding perspective to signify the durations of ongoing stimuli. Sustained responses might also facilitate the detection of temporally overlapping novel stimuli [24, 34].

Our observation that onset responses contain stimulus-specific information is consistent with behavioral results indicating that sounds of extremely short durations (as short as 3 ms) are recognizable [35–39]. The relationship between tuning curve width and information encoding has been the focus of many theoretical discussions [40–43]. Our result is consistent with theoretical analysis showing that response selectivity and the quality of sensory information encoding does not have a definitive relationship [44]. It should be noted that due to the necessary acoustic transients at the beginning and end of each sound and in spite of the ramps applied to the sounds’ amplitude envelopes, the sound stimuli contain other frequency components in addition to the tonal frequencies at these transients. The particular effects of these other sound frequencies on neural activities are not explicitly considered in the current study. The much weaker energy of these frequencies and the observation that central auditory circuits exist to improve spectral resolution in the face of broad acoustic transients [45] potentially mitigate the effects of these other frequencies in the transients. To gain more insight into this question, one could potentially vary the duration of the tone onset ramps to study the effects of spectral splatter on neural encoding. Prior studies have shown that single neurons could provide temporally precise spiking code for the encoding of slope [46] and duration [47, 48] of onset ramps. It would also be important to determine whether the sound encoding strategies proposed in this study hold for more complex sounds such as speech that varies in temporal envelope. For example, different neural codes are shown to exhibit different correlations in the decoding performance relative to behavioral performance for consonant and vowel discriminations [49].

Another caveat of the current study is that neural activities were sequentially recorded and aggregated offline to compose the neural population responses instead of simultaneously measured for the entire neural population. Although similar methods have been widely used and shown effective for studies of neural population responses [11, 23–25, 29], sequential sampling does not provide information on the noise correlations of neuronal spiking activities, which might play an important role in information encoding in neural systems [15, 42, 50]. How noise correlations evolve over time and affect information encoding across the onset and sustained response epochs is an important topic for future investigations. An investigation of neuronal response correlations in primate visual cortex has revealed that rapid adaptation to the statistical properties of the preceding stimuli altered the noise correlation profile across the neural population to the subsequent stimuli and improved the coding efficiency for stimulus discrimination [51]. It remains to be seen whether similar effects exist for the adaptation occurring during the presentation of a single stimulus. It is possible that trial-by-trial simultaneous recordings that take into account noise correlation effects would alter the current results.

The relationship we have described between neural activity amplitude and neural encoding reliability is consistent with the analysis of Poisson spiking behavior using a Bayesian framework [52, 53]. Because uniformly adaptive Poisson-like population spiking behavior is sufficient to give rise to the constant spike count encoding phenomenon, sensory systems may employ this strategy generally. Neural decoders appear to be able to take on a wide variety of forms and still extract equivalent stimulus-specific information for any constant spike count. It is worth pointing out that the Poisson rate code is one of the simplest neural codes. Other forms of neural coding using higher order correlations between neurons’ spiking activities, for example, might convey more information with fewer spikes.

## Materials and methods

### Ethics statement

All procedures were approved by the Institutional Animal Care and Use Committee of Washington University in St. Louis in protocol 20130242. Washington University utilizes the Guide for the Care and Use of Laboratory Animals, the Animal Welfare Act, PHS policy, and US Government Principles for the Utilization and Care of Vertebrate Animals Used in Testing, Research and Training for all research involving animals.

### Electrophysiological recordings

Neural activity was recorded from A1 of 2 marmoset monkeys (*Callithrix jacchus*). Details of the recording technique were reported previously [54]. Briefly, a one-time surgery was performed to implant restraining head posts and expose the skull over the recording areas. Animals were allowed to fully recover from surgery before the first recording experiment started. During recordings, animals sat quietly in the awake state in a custom-made primate chair with head fixed inside a double-wall, sound-proof booth (IAC 120a-3, Bronx, NY). Animals were acclimated to the chair prior to head post implantation.

High-impedance single tungsten microelectrodes (approximately 5 MOhms at 1 kHz; FHC, Inc, Bowdoin, ME) were used for recording extracellular neural activity. Electrodes were advanced into the cortex through micro-craniotomy holes (0.8 mm in diameter) drilled over A1; the location was determined based on the location of lateral sulcus and measurements guided by a standard marmoset brain atlas [55]. Location was confirmed by the topographical mapping of the neurons’ best frequencies [56]. During recording, electrodes were gradually lowered into the cortex by the experimenter via a hydraulic micromanipulator (FHC, Inc, Bowdoin, ME). The recorded signals were first amplified and filtered (AM Systems 1800, Sequim, WA), then fed into an online template-based spike sorting software (Alpha-Omega, Nazareth, Israel) to separate simultaneously detected neurons. Single and multi units were determined initially through visual inspection of the action potential waveforms. Sufficient amplitude, distinct shape, and small variation across individual spikes were features used to identify single units. Inter-spike interval distributions were quantified to confirm the results. All single units had no more than 0.45% spikes with less than 1 ms inter-spike intervals. For simplicity of interpretation of the results, only verifiable single units were included in the analysis, although all results and conclusions held when multi units were included (results not shown). All single units isolated during the experimental period were included for analysis (i.e., no exclusion criteria). Median signal-to-noise ratio of recorded spikes using this procedure is 24.5 dB.

### Sound stimuli

Sound stimuli were delivered with a free field speaker (B&W 601S3, Worthing, UK) placed exactly 1 meter in front of the animal. A broad range of sounds was typically used to search for neurons, including pure tones of different frequencies and intensities, random spectrum stimuli with different parameters [6], amplitude or frequency modulated pure tones, clicks, white noise, and marmoset vocalizations. Neurons were tested with the experimental stimuli and included in the analysis as long as they demonstrated subjective responses to any of the searching stimuli. In other words, the primary data set includes all auditory A1 single units encountered in either hemisphere of the 2 monkeys regardless of responsiveness to the actual experimental stimuli.

For each identified auditory neuron, responses to a set of pure tones from 1 to 32 kHz, 1 octave apart (i.e., 1, 2, 4, 8, 16, 32 kHz) at 85 dB SPL were elicited. The rationale for this selection of tones was to ensure that a sufficient number of neurons could be collected across the full range of hearing to represent the information in the stimulus set, while providing some instances of receptive field overlap and others with none. The intent of this study was to examine across the full frequency range of hearing the prediction that sustained spiking contains more stimulus information than onset spiking, as well as to compare the speed with which information is conveyed during different epochs.

The stimulus set was repeated pseudorandomly 20 times. Each tone was 500 ms long with 5 ms on and off ramps. In each trial, recording began 300 ms before stimulus onset and ended 700 ms after stimulus offset. The inter-stimulus interval was at least 1 second. Altogether, 171 single units were recorded from both hemispheres of the experimental animals.

### Stimulus-induced spiking rate and single-neuron selectivity

The spiking rate of each neuron in response to each stimulus was calculated in overlapping windows throughout the recording. Unless otherwise noted, windows measured 15 ms wide with 5-ms displacement between adjacent windows. The spiking rates of all neuron-stimulus pairs were then averaged to reflect the main dynamics of the neural responses over time (Fig 1A).

Selectivity of a neuron was quantified over time by the number of stimuli in the stimulus set to which the neuron showed significant increase in spiking rate compared to the spontaneous spiking rate at that time point: the larger this number, the lower the selectivity. Poisson conditional testing [57] was used to test for a significant increase in spiking rate, with null hypothesis that the spiking rate in a window was equal to or smaller than the spontaneous spiking rate measured before stimulus onset. Windows of 50 ms length with 1-ms displacement between adjacent windows throughout the recording were tested. Bonferroni correction was used to correct for multiple comparisons. Windows with *P* values after correction smaller than 0.05 were regarded as containing significant responses.

### Mutual information between stimuli and neural responses

For each neuron the mutual information between the stimulus set and its responses was calculated in each 15-ms window throughout the recording with 5-ms displacement between neighboring windows. Mutual information *I*(*n*,*s*) was quantified as the total entropy *H*_*T*_(*n*) of the spike count distribution across all stimuli minus the mean of the entropy conditioned upon each stimulus *H*_*i*_(*n*|*s*):

$$\text{Mutual}\;\text{information}:\;I(n,s)=H_{T}(n)-\frac{1}{N}\sum_{i}H_{i}(n|s)\text{,}$$ (1)

where *N* is the number of stimuli, equal to 6 in this case. Specifically, total entropy and conditional entropy are defined as follows:

$$\text{Total}\;\text{entropy}:\;H_{T}(n)=\sum_{k}p_{k}^{total}\text{log}(p_{k}^{total})$$ (2)

$$\text{Conditional}\;\text{entropy}:\;H_{i}(n|s)=\sum_{k}p_{k}^{i}\text{log}(p_{k}^{i}),$$ (3)

in which $p_{k}^{total}$ and $p_{k}^{i}$ are the probability of firing *k* spikes in the distribution across all stimuli and the distribution for the *i*^*th*^ stimulus, respectively [18]. In actual calculations, probabilities $p_{k}^{total}$ and $p_{k}^{i}$ are approximated by the frequency of occurrence.

To correct for the finite data bias in calculating entropies, the bias correction method proposed by Treves and Panzeri [19] was employed, which has been widely used in the calculation of information content for neural responses [18, 20]. Specifically, the relationship between the entropy $H_{\text{exp}}^{M}$ estimated from a limited sample size *M* and the true entropy *H*_*true*_ can be approximated to the second-order correction terms:

$$H_{\text{exp}}^{M}=H_{true}+\frac{c_{1}}{M}+\frac{c_{2}}{M^{2}}\text{.}$$ (4)

A linear regression was used to fit the entropy estimates as a function of sample size. The true entropy was then obtained as the ordinate intercept of the best-fit function. Specifically, entropy was calculated from data sets of sizes from 10% to 100% of the original data sets with 10% step size. For every data size, 100 random data sets were drawn and the average entropy value was fed into the linear regression equation.

Eq 4 fails to capture the difference between the true entropy and estimated entropy when the sample size is too small. To check against excessive finite data bias, the Ma bound was calculated for each entropy condition [18, 20]. Ma bound is a lower bound for the response entropy and is less biased by limited sample size:

$$H_{Ma}=-{\text{log}}_{2}(\langle p_{k}\rangle )=-log_{2}(\sum_{k=0}^{\infty }p_{k}^{2}).$$ (5)

For all the entropy conditions in our case, the Ma bound was smaller than the entropy directly estimated using all samples, indicating sufficient data for entropy calculation in our data set.

Note that following the step of bias correction, the resulting value of the mutual information could be negative. The magnitudes of the negative values were much smaller compared to the range of positive values (<6%). No special processing was applied to these negative values during the calculation, including the calculation of the mean and quantile of the information rates.

### Decoding stimulus identity with single neuron responses

The accuracy of decoding stimulus identity from single neuron responses over time was tested in 15-ms windows with 5-ms displacement between adjacent windows. A single-trial neural response is represented as the spiking rate of the neuron derived from its spike count in that trial falling within the analysis window. A template-based classification procedure was used for the decoding, in which the entire data set was first divided into training and testing data sets. The mean spiking rate for each stimulus was obtained from the training data set as the template for that response. Each response in the test data set was then classified into the stimulus whose response template is the closest to the test response. A 4-fold cross validation procedure was used. The average decoding accuracy was quantified as the mean percentage of correctly classified responses.

### Neural population trajectory analysis

To reveal the neural population response dynamics, concepts from linear algebra and dynamical systems theory were used to develop population response trajectories [11, 23, 24, 26]. Responses of each neuron-stimulus pair were first binned into 10-ms nonoverlapping windows throughout recording, and mean spiking rate in each window was obtained by averaging across the repetitively tested trials. Neural population responses were formed by pooling the sequentially collected neurons in the data set. Spiking rate responses of all neurons to the same stimulus were aligned at sound onset. The population neural response in each window was represented as a high-dimensional vector with the spiking rate of each neuron as one dimension. The complete population response at this point was composed of a time series of vectors in the high-dimensional response space.

Geometrically, a population response vector can be represented as a single point in the high-dimensional neural response space. Connecting the vector values in temporal order then forms a trajectory that reflects the population response dynamics. To obtain an intuitive visualization of the population response dynamics, a low-dimensional representation of the trajectories was obtained by applying principal component analysis (PCA) to project the trajectories into the coordinates that capture the greatest amount of variance of the data. To facilitate visual inspection, only responses from 30 ms before sound onset to sound offset were included in this analysis (53 individual nonoverlapping 10-ms windows). In applying PCA, responses of different stimuli were concatenated, and thus a matrix of 171 rows (corresponding to 171 neurons) and 318 columns (corresponding to 6 stimuli and 53 windows for each stimulus) was formed. PCA was performed to project the 318 samples into a new set of coordinates over the 171 dimensional space that sequentially carried the most variance of the data set. The trajectories were then smoothed by 5-point averages and plotted on axes corresponding to the first 3 principal components.

### Population neural decoding

To directly assess the quality of sound encoding for neural responses in a given time window, decoders were constructed to recover the stimulus identity from a single-trial response of the neural population, thereby evaluating decoding accuracy. Neural population responses were formed in the same way as in trajectory analysis, except that spiking rates for each neuron in this case were extracted from a single trial instead of averaged over trials, and different window lengths were used depending on the purpose of the analysis. Decoders in different windows were built separately and independently from one another. A classification scheme was used to build the decoders, in which the parameters of the decoder were determined from the training set and the performance of the decoder was tested on the testing set. Specifically, a 4-fold cross validation procedure was used, in which 15 out of the 20 repetitively tested trials in the data set were used to form the training set in turn and the remaining 5 trials for each stimulus composed of the testing set. The mean percentage of correctly decoded trials was then used as the decoding accuracy.

To avoid potential bias from any one particular type of decoder, 3 different decoding strategies were evaluated whenever decoding was used to assess the neural responses. The first type of decoder was constructed with LDA. To do so, the dimensionality of the population response was first reduced with PCA. In this case, each neuron represented one dimension, and each trial of neural responses in the training set represented one sample. The number of principal components accounting for 90% of the data’s variance was retained. These data were then fed into LDA, a technique that linearly projects the data onto a few dimensions that maximize the ratio of between-class variance to within-class variance for the data set. The mean response to each stimulus was then obtained from the training set. During testing, a test sample was first projected with the same PCA and LDA transformations determined from the training data set. The sample was then classified into the stimulus group whose mean response was closest to it. The distance of the test sample **r**_*test*_ to the mean response **r**_*mean*_ of each stimulus was calculated as in Eq 6, where the symbol ‖‖ denotes the length of a vector or the square root of its *L*^2^ norm:

$$d=\left\|{\text{r}}_{mean}-{\text{r}}_{test}\right\|\text{.}$$ (6)

The other 2 methods were both based upon template matching but with different distance metrics. In this procedure, a template for responses to each stimulus was first obtained as the mean response to the stimulus in the training set. During testing, the distance of the test sample to each template was measured, and the sample was classified to the group whose template was closest. Given template **r**_*a*_ and test sample **r**_*test*_, the Euclidean distance *d*_*Euclidean*_ between them is defined as in Eq 7. The angular distance *d*_*angular*_ is defined as in Eq 8, where the symbol 〈 〉 denotes the inner product of 2 vectors.

$$d_{Euclidean}=\left\|r_{a}-r_{test}\right\|$$ (7)

$$d_{angular}=1-\frac{\langle r_{a},r_{test}\rangle }{\left\|r_{a}\right\|\left\|r_{test}\right\|}\text{.}$$ (8)

### Assessing the effect of window length on decoding accuracy

For this purpose, decoding analysis was performed in windows with the same beginning time but extended to different ending time. Window lengths from 2 to 150 ms were tested in 2-ms step size for windows beginning at either 20 ms or 200 ms after sound onset (Fig 3). These 2 time points are chosen as examples for the onset and sustained response epoch, respectively, because they are relatively early within each epoch allowing better analysis with longer window length. They were identified as belonging to each epoch based on the visual inspection of the overall spiking rate curve in Fig 1A. The exact decoding results for each condition also depend on the specific split of training and testing data sets. To evaluate this uncertainty, an additional analysis was performed, in which instead of using 4-fold cross validation, decoding accuracies were evaluated for each of 1,000 runs with random splits of the training and testing set. Specifically, 15 out of the 20 trials were randomly selected as the training set, and the remaining 5 trials were used for testing (S2 Fig). The 3 decoding methods were evaluated separately, each with 1,000 random splits of the data set. It should be noted that decoding accuracies using different splits of the same data set are not independent measurements. In addition, because the subject of the investigation is the performance of a neural population, the study contains measurements of only one such example. Results in S2 Fig should not be mistaken for measurements from multiple neural populations.

To provide a complete picture for results at different time points, windows beginning at 0 to 400 ms after sound onset were tested in 5-ms step size for window length ranging from 0 to 200 ms in 1-ms step size. A saturation decoding accuracy was obtained for each window beginning time as the mean decoding accuracy for window length between 150 to 200 ms (Fig 4A–4C). Then the minimum window length at which the decoding accuracy was equal to or above 80% of the saturation accuracy was obtained for each window beginning time (Fig 4D–4F).

### Decoding accuracy with predetermined spike counts

Decoding analysis was performed in analysis windows containing a specified number of spikes (Fig 5, S3 and S4 Figs). To determine the proper length of an analysis window, its left edge was fixed and the window was elongated to the right until the desired population spike count had been accumulated. The spike count was obtained as the total number of spikes across all neurons in all 20 trials to all stimuli in the window. After the length of the window was decided, the population neural decoding accuracy was then assessed using all 3 decoders described above. Windows were placed such that the left edges of 2 neighboring windows were 5 ms apart.

### Fano factor

The Fano factor was calculated for neural responses in 15-ms nonoverlapping windows throughout the recording for every neuron to each stimulus. Spike count in the window was obtained for each repetitively tested trial. The variance and the mean of the spike counts across trials were then calculated. The Fano factor was obtained as the variance of the spike count divided by the mean.

### Quantifying reliability of spiking rate estimation

The CV was used to quantify the reliability of a neuron’s spiking activity. Due to the trial-to-trial variability of neural responses, the number of spikes a neuron fires in a time window varies across repetitive tests. The spiking rate derived from the spike count is thus a random variable. CV is the ratio of the standard deviation over the mean for this spiking rate estimate. It measures the absolute variability of the spiking rate relative to its true magnitude, thus offering a quantification more closely related to the informational capacity of the neural activity than the absolute variability value. In reality, the standard deviation and the mean of the spiking rate estimate are calculated from the repetitively tested trials. For Poisson neurons, the CV and the true spiking rate have a fixed relationship given a fixed window length. To examine the relationship between these 2 values in real neural data, the mean spiking rate was used as an estimate of the true rate. CV and the mean spiking rate were calculated for each neuron and stimulus pair in 15-ms nonoverlapping windows. The relationship between the CV and the mean spiking rate was then compared to that of Poisson neurons (Fig 6B). To quantify how the observed relationship approaches that of Poisson-spiking neurons, the coefficient of determination (*r*^2^) between theoretical CV values *CV*_*Poisson*_(*i*) and observed CV values *CV*_*obs*_(*i*) was calculated. *CV*_*Poisson*_(*i*) was calculated by Eq 9 as derived in S1 Text, in which *r*_*m*_(*i*) is the corresponding mean spiking rate:

$$CV_{Poisson}(i)=\frac{1}{\sqrt{r_{m}(i)T}}\text{.}$$ (9)

The mean of the observed CV value was then calculated by Eq 10. The coefficient of determination then assessed the amount of explained variance in the data set (Eq 11).

$$\bar{CV}=\sum_{i}CV_{obs}(i)$$ (10)

$$r^{2}=1-\frac{\sum_{i}{\left(CV_{obs}(i)-CV_{Poisson}(i)\right)}^{2}}{\sum_{i}{\left(CV_{obs}(i)-\bar{CV}\right)}^{2}}$$ (11)

### Simulation

To investigate how the sound encoding capacity of ideal Poisson neurons changes with their spiking rates, a population of purely Poisson-spiking neurons was simulated with spiking rates derived from the experimental data. Specifically, the spiking rates of real neurons were calculated in the duration of 30 to 38.8 ms after sound onset. This is also a window containing 2,000 spikes across all neurons and trials of all stimuli. To simulate the responses of a Poisson-spiking neuron with the spiking rate of a real neuron *p* to stimulus *q*, the spiking rate of the real neuron $r_{p}^{q}$ was multiplied by coefficient $k_{r}^{i}$ and the read-out window length *T* was multiplied by coefficient $k_{T}^{i}$. The expected spike count of the Poisson-spiking neuron was then calculated as $k_{r}^{i}r_{p}^{q}k_{T}^{i}T$. Note that $k_{r}^{i}$ and $k_{T}^{i}$ are the same for all neurons in the same simulated population. The spike count of each neuron was generated as a random number from a Poisson distribution with mean equal to $k_{r}^{i}r_{p}^{q}k_{T}^{i}T$. The responses were simulated for 20 trials, and the decoding was performed for the simulated populations by using the same 3 decoders as for real neurons. To simulate the decoding accuracy of uniformly adapted neurons (Fig 6C), $k_{r}^{i}$ was varied in the range [0.1–1] in 0.1 step sizes, and $k_{T}^{i}$ was set to $\frac{1}{k_{r}^{i}}$ to maintain the expected spike count. To simulate the effect of spiking rate magnitude on decoding accuracy (Fig 6D), $k_{r}^{i}$ was varied in the range [0.1–1.5] in 0.1 step sizes, and $k_{T}^{i}$ was maintained at 1.

### Extensive testing of the constant spike count encoding with neuron subsets

To ensure an adequate assessment of the relationship between neuronal selectivity and decoding ability, a subset of neurons was selected containing all the neurons with individually lower selectivity during the onset-response epoch than the sustained-response epoch and responded to at least 1 sound during the sustained epoch. To do so, the results of neuronal selectivity quantification above was used, in which for each neuron, the number of stimuli it actively responded to was determined in 1-ms step size. Then the following 2 criteria were used to select the subset of neurons: (1) the maximum number of stimuli a neuron responded to during the first 200 ms after sound onset was larger than the maximum number of stimuli it responded to during the last 300 ms of sound presentation (note that the sound stimuli were 500 ms long, and (2) the median number of stimuli a neuron responded to during the last 300 ms of sound presentation was equal to or greater than 1. The decoding analysis with windows containing the same number of spikes was then repeated with this subset of neurons, and also repeated with the rest of the neurons in the population excluding this subset.

### Preliminary analysis of decoding accuracy among tones of finer frequency differences

To gain some insights into the information content of neural responses on tones of finer frequency differences, previously recorded neuronal tone-response data were aggregated and evaluated for decoding accuracy. The first additional data set consisted of the responses of 478 neurons stimulated with 9 pure tones between 8 to 16 kHz at 1/8 octave frequency spacing. The second additional data set consisted of a subset of 376 neurons from the first data set stimulated with 17 tones between 8 to 16 kHz at 1/16 octave frequency spacing. These 2 data sets contained all neurons tested with the indicated stimuli from 13 hemispheres of 9 animals. For both data sets, tonal stimuli were 100 ms long, and subsequent tones were separated by at least 500 ms of silence. Recording techniques were the same as described above. Neural responses were recorded in each trial from 200 ms before sound onset to 300 ms after sound offset. A total of 5 trials were recorded for each sound. The same 3 types of decoders as described above were used for decoding tone identity from neural responses. Decoding accuracy was assessed in 15-ms and 50-ms windows with 5-ms step size between adjacent windows. To compare the trend of decoding accuracy change with time for decoding among tones with different frequency spacing, the decoding accuracy in each condition was self-normalized by dividing the standard deviation of the decoding accuracy across time from 200 ms before sound onset to 100 ms after sound onset, and then subtracting an offset so that the mean decoding accuracy during the 200 ms before sound onset was zero (S6 Fig).

## Supporting information

S1 Fig Decoding accuracy across time under 3 different window lengths. Accuracy of decoding sound identity with single-trial neural population responses at different time points is shown for 8-ms window size in red. Results with 15- and 50-ms windows from Fig 2B–2D are also shown to facilitate comparison. Figure formats are the same as in Fig 2B–2D. Numerical data and analytical results can be found at osf.io/xhmus/.
(TIF)

S2 Fig Uncertainty from random splits of training and testing set for decoding under different window lengths. Figure formats are the same as Fig 3A–3C. Solid curves show the mean decoding accuracy across 1,000 runs with random splits of the training and testing data sets. Error bars show 1 standard deviation above and below the mean. Results for windows starting at 200 ms were plotted in partial transparency to allow visualization of overlapping figure elements. Numerical data and analytical results can be found at osf.io/xhmus/.
(TIF)

S3 Fig Constant spike count decoding using neuron subset with the broadest tuning. Figure formats are the same as Fig 5, except that window construction and decoding in this case was performed using the subset of neurons with lower selectivity during the onset response epoch and responded to at least 1 sound during the sustained response epoch. Numerical data and analytical results can be found at osf.io/xhmus/.
(TIF)

S4 Fig Constant spike count decoding with the entire population excluding the subset with the broadest tuning. Figure formats are the same as Fig 5, except that window construction and decoding was performed with the entire population excluding the subset of neurons used in S3 Fig. Numerical data and analytical results can be found at osf.io/xhmus/.
(TIF)

S5 Fig Decoding accuracy among tones with finer frequency differences. (A-C) Accuracy of decoding among 9 tones between 8 to 16 kHz with 1/8 octave frequency spacing, using a data set of 478 neurons. (D-F) Accuracy of decoding among 17 tones between 8 to 16 kHz with 1/16 octave frequency spacing, using a data set of 376 neurons. Results of decoding with 15- and 50-ms windows are shown in gray and black, respectively. Tones were 100 ms long. To facilitate comparison, abscissa was plotted in the same scale and range as in Fig 2B–2D. Numerical data and analytical results can be found at osf.io/xhmus/.
(TIF)

S6 Fig Comparison of the change of decoding accuracy with time for decoding tones of different frequency spacing. Normalized decoding accuracies for decoding tones with 3 different frequency spacings (1, 1/8, 1/16 octave) are plotted in blue, green and red, respectively. (A-C) shows the results of decoding using 15-ms windows with 3 different decoders. (D-F) shows the results of decoding using 50-ms windows with 3 different decoders. Numerical data and analytical results can be found at osf.io/xhmus/.
(TIF)

S1 Text Spiking variability of Poisson neurons. (DOCX)

S2 Text The effect of uniform adaptation on the discriminability of Poisson spiking neurons. (DOCX)

## Abbreviations

A1: primary auditory cortex
CV: coefficient of variation
LDA: linear discriminant analysis
PCA: principal component analysis
SPL: sound pressure level
TMPCOS: template matching based on an angular distance metric
TMPEUC: template matching based on a Euclidean distance metric
