# Supplementary figures and images for: Rate, not selectivity, determines neuronal population coding accuracy in auditory cortex
Source: PLoS Biol. 2017 Nov 1;15(11):e2002459. doi: 10.1371/journal.pbio.2002459 (PMC5683657; doi:10.1371/journal.pbio.2002459)

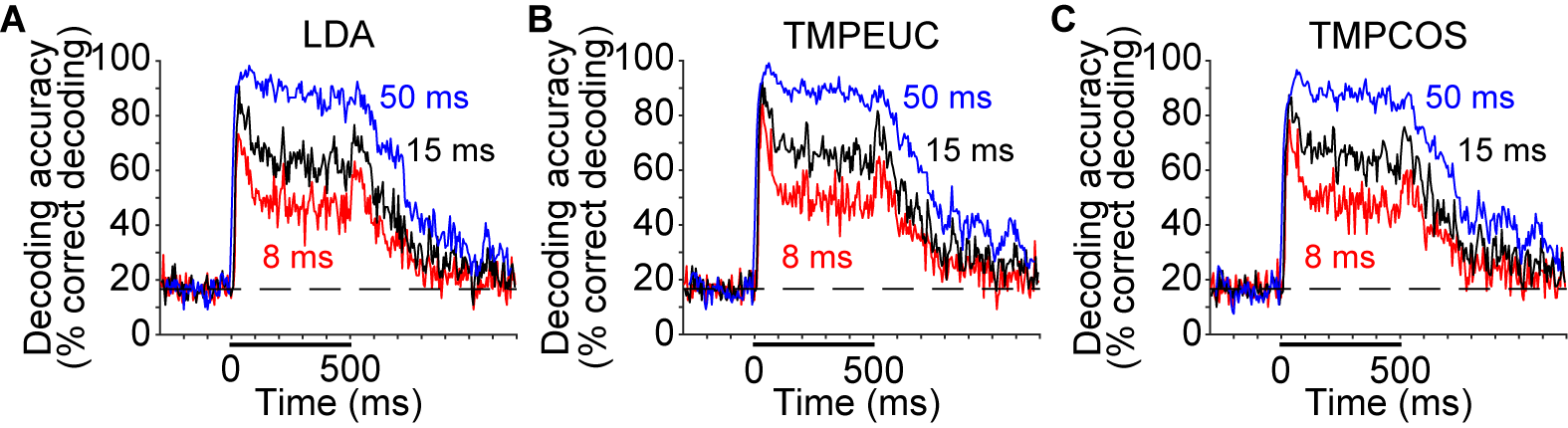

Supplement: S1 Fig — Accuracy of decoding sound identity with single-trial neural population responses at different time points is shown for 8-ms window size in red. Results with 15- and 50-ms windows from Fig 2B–2D are also shown to facilitate comparison. Figure formats are the same as in Fig 2B–2D. Numerical data and analytical results can be found at osf.io/xhmus/. (TIF) [file pbio.2002459.s001.tif]

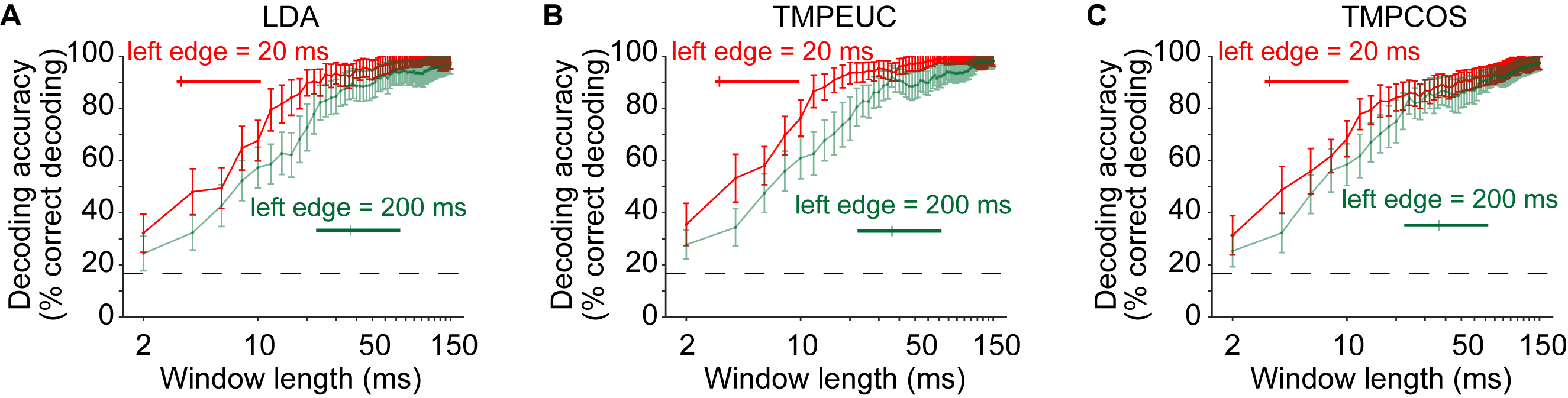

Supplement: S2 Fig — Figure formats are the same as Fig 3A–3C. Solid curves show the mean decoding accuracy across 1,000 runs with random splits of the training and testing data sets. Error bars show 1 standard deviation above and below the mean. Results for windows starting at 200 ms were plotted in partial transparency to allow visualization of overlapping figure elements. Numerical data and analytical results can be found at osf.io/xhmus/. (TIF) [file pbio.2002459.s002.tif]

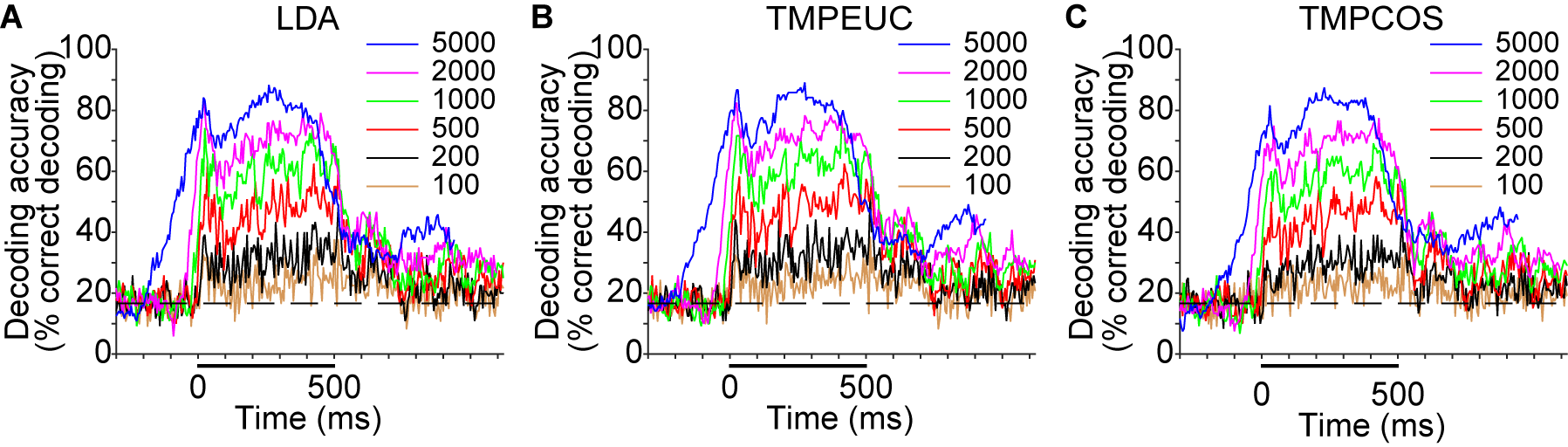

Supplement: S3 Fig — Figure formats are the same as Fig 5, except that window construction and decoding in this case was performed using the subset of neurons with lower selectivity during the onset response epoch and responded to at least 1 sound during the sustained response epoch. Numerical data and analytical results can be found at osf.io/xhmus/. (TIF) [file pbio.2002459.s003.tif]

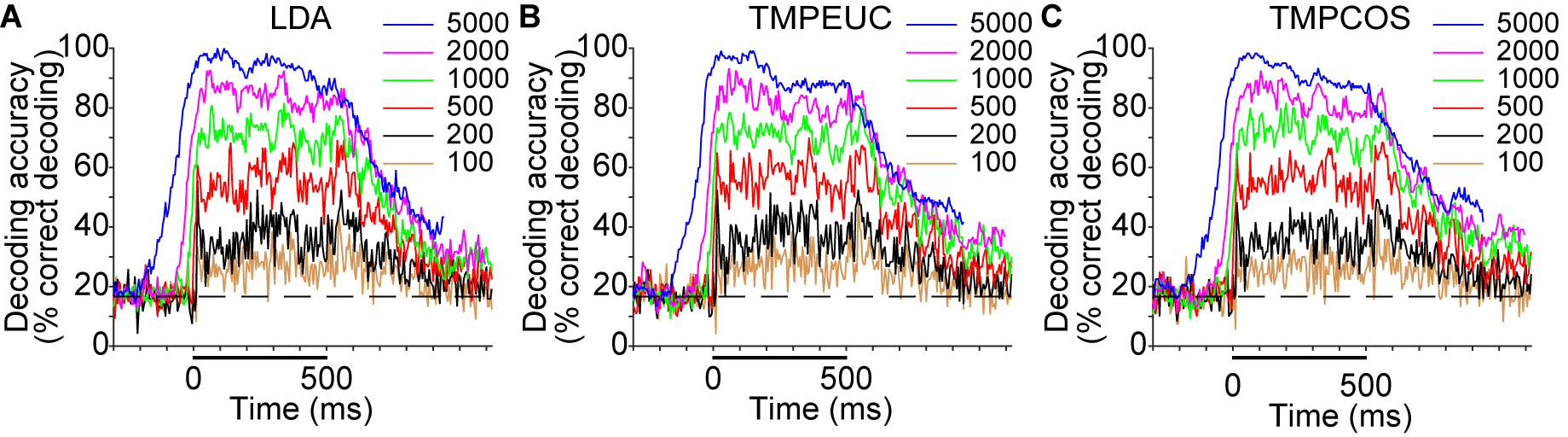

Supplement: S4 Fig — Figure formats are the same as Fig 5, except that window construction and decoding was performed with the entire population excluding the subset of neurons used in S3 Fig. Numerical data and analytical results can be found at osf.io/xhmus/. (TIF) [file pbio.2002459.s004.tif]

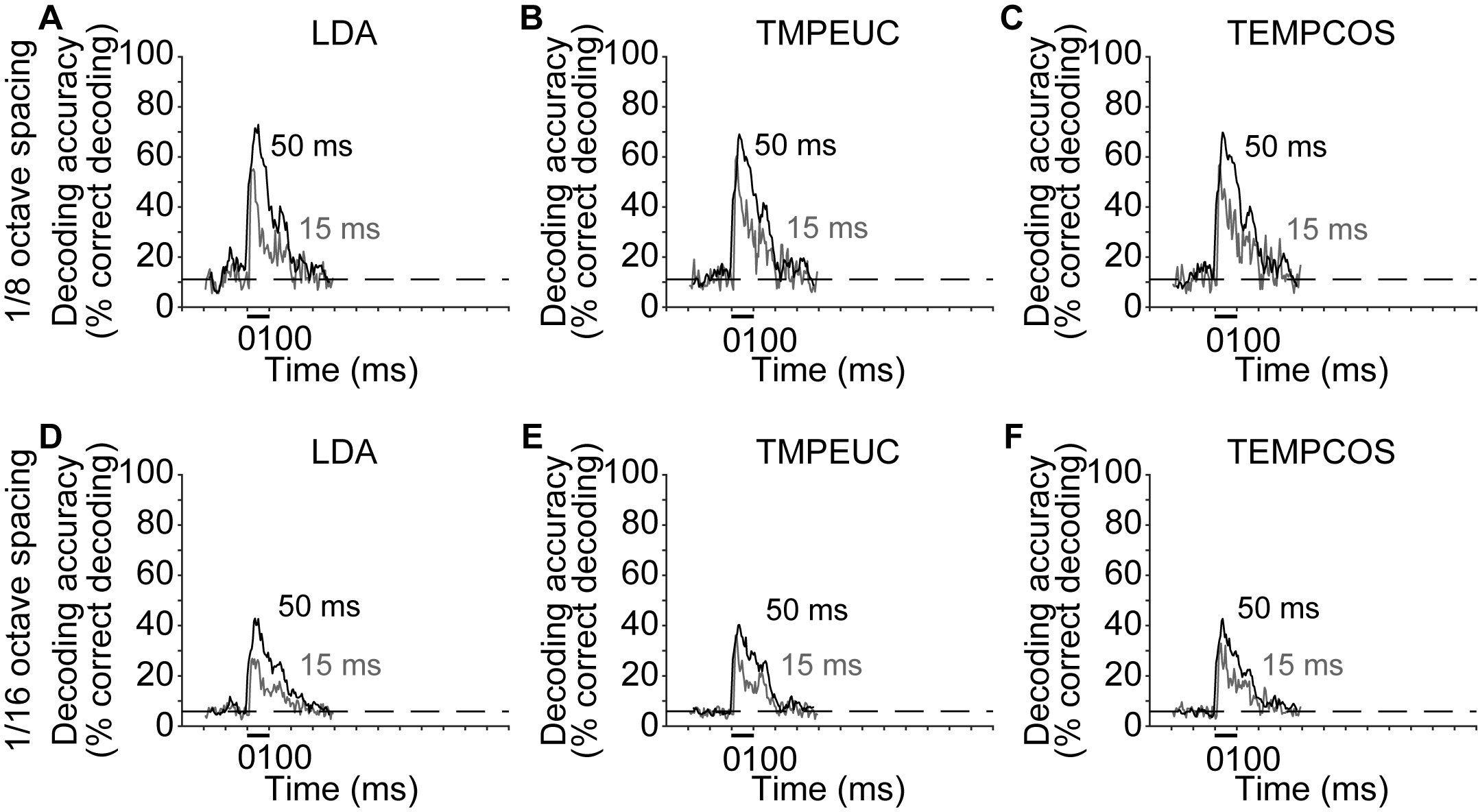

Supplement: S5 Fig — (A-C) Accuracy of decoding among 9 tones between 8 to 16 kHz with 1/8 octave frequency spacing, using a data set of 478 neurons. (D-F) Accuracy of decoding among 17 tones between 8 to 16 kHz with 1/16 octave frequency spacing, using a data set of 376 neurons. Results of decoding with 15- and 50-ms windows are shown in gray and black, respectively. Tones were 100 ms long. To facilitate comparison, abscissa was plotted in the same scale and range as in Fig 2B–2D. Numerical data and analytical results can be found at osf.io/xhmus/. (TIF) [file pbio.2002459.s005.tif]

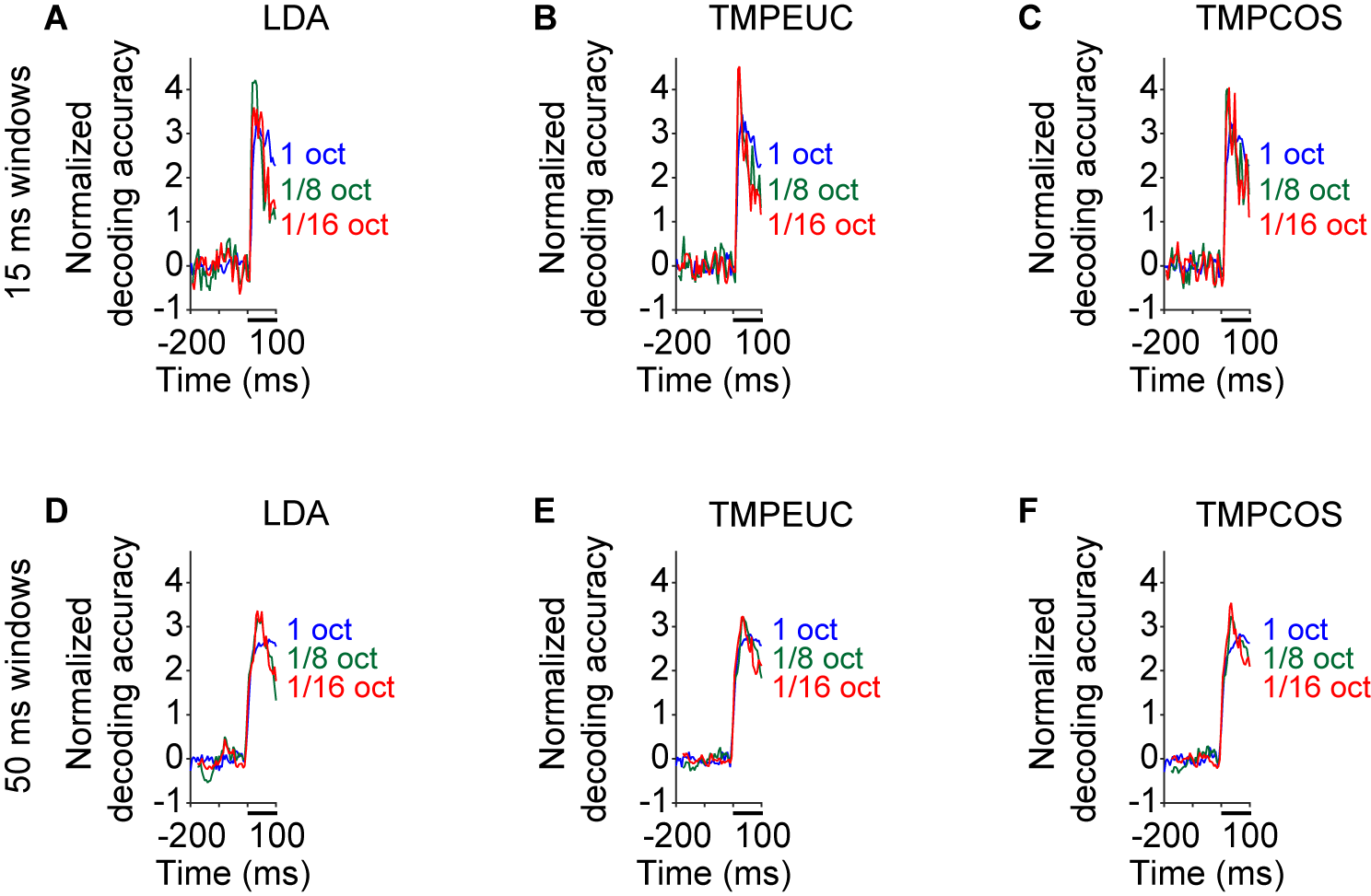

Supplement: S6 Fig — Normalized decoding accuracies for decoding tones with 3 different frequency spacings (1, 1/8, 1/16 octave) are plotted in blue, green and red, respectively. (A-C) shows the results of decoding using 15-ms windows with 3 different decoders. (D-F) shows the results of decoding using 50-ms windows with 3 different decoders. Numerical data and analytical results can be found at osf.io/xhmus/. (TIF) [file pbio.2002459.s006.tif]
