# Supplementary material for: Rate, not selectivity, determines neuronal population coding accuracy in auditory cortex
Source: PLoS Biol. 2017 Nov 1;15(11):e2002459. doi: 10.1371/journal.pbio.2002459 (PMC5683657; doi:10.1371/journal.pbio.2002459)
Supplement: S1 Text — (DOCX) [file pbio.2002459.s007.docx]

S1 Text: Spiking variability of Poisson neurons

For a Poisson spiking neuron with true spiking rate $r_{true}$, the number of spikes it fires in an interval $T$ is a random variable $n$ following a Poisson distribution, whose mean and variance are both equal to $r_{true}T$. Estimating the neuron’s spiking rate from its spike count gives the spiking rate estimate $r$:

$r=\frac{n}{T}\text{ (12)}$.

The mean and standard deviation of the spiking rate estimate can then be calculated as follows.

$$\mathrm{E}(r)=\frac{\mathrm{E}(n)}{T}=\frac{r_{true}T}{T}=r_{true}\text{ (13)}$$

$\mathrm{Std}(r)=\sqrt{\mathrm{Var}(r)}=\sqrt{\frac{\mathrm{Var}(n)}{T^{2}}}=\sqrt{\frac{r_{true}T}{T^{2}}}=\sqrt{\frac{r_{true}}{T}}\text{ (14)}$.

This leads to the coefficient of variation (CV) in (15), where CV is determined entirely by the expected number of spikes in the interval:

$CV(r)=\frac{\mathrm{Std}(r)}{\mathrm{E}(r)}=\frac{\sqrt{\frac{r_{true}}{T}}}{r_{true}}=\frac{1}{\sqrt{r{}_{true}T}}\text{ (15)}$.
