# Supplementary material for: Rate, not selectivity, determines neuronal population coding accuracy in auditory cortex
Source: PLoS Biol. 2017 Nov 1;15(11):e2002459. doi: 10.1371/journal.pbio.2002459 (PMC5683657; doi:10.1371/journal.pbio.2002459)
Supplement: S2 Text — (DOCX) [file pbio.2002459.s008.docx]

S2 Text: The effect of uniform adaptation on the discriminability of Poisson spiking neurons

Considering a Poisson spiking neuron with mean spiking rate $r_{1}$ and $r_{2}$ to two stimuli, the discriminability of the neuron between the two stimuli can be quantified by $d'$ as in equation (16), where $\sigma_{1}^{2}$ and $\sigma_{2}^{2}$ are the variance of the spiking rate estimate obtained from its spike count:

$d'=\frac{r_{1}-r_{2}}{\sqrt{\frac{1}{2}(\sigma_{1}^{2}+\sigma_{2}^{2})}}\text{ (16)}$.

Following the relationship between the spiking rate estimate and the spike count in equation (12), $\sigma_{1}^{2}$ and $\sigma_{2}^{2}$ can be obtained as follows:

$$\begin{matrix} \sigma_{1}^{2}=\frac{\mathrm{Var}(n_{1})}{T^{2}}=\frac{r_{1}T}{T^{2}}=\frac{r_{1}}{T}\text{ (17)} \\ \sigma_{2}^{2}=\frac{\mathrm{Var}(n_{2})}{T^{2}}=\frac{r_{2}T}{T^{2}}=\frac{r_{2}}{T}\text{ (18).} \end{matrix}$$

This gives rise to an expression of $d'$ in terms of $r_{1}$ and $r_{2}$, as well as the observation window length $T$:

$d'=\frac{r_{1}-r_{2}}{\sqrt{\frac{1}{2}(\frac{r_{1}}{T}+\frac{r_{2}}{T})}}=\frac{r_{1}-r_{2}}{\sqrt{\frac{1}{2T}(r_{1}+r_{2})}}\text{ (19)}$.

In the situation of uniform adaptation, the mean spiking rates to the two stimuli are adapted by the same ratio. The spiking rates after adaptation can be denoted by $kr_{1}$ and $kr_{2}$. The question at hand is whether after uniform adaptation the neuron would maintain the same discriminability to the two stimuli, given an observation window allowing the same expected spike count. With decreased spiking rate, the observation window needs to be proportionally elongated to maintain the same expected spike count. The observation window length then becomes $\frac{T}{k}$. Substituting the spiking rate and observation window length from equation (19), the discriminability of the neuron after uniform adaptation $d'_{adapted}$ is given in equation (20). With some simple manipulation, it can be seen that the discriminability of the neuron after uniform adaptation equals that before adaptation:

$$d'_{adapted}=\frac{kr_{1}-kr_{2}}{\sqrt{\frac{1}{2\frac{T}{k}}(kr_{1}+kr_{2})}}=\frac{k(r_{1}-r_{2})}{\sqrt{\frac{k^{2}}{2T}(r_{1}+r_{2})}}\text{ (20)}$$

$d'_{adapted}=\frac{r_{1}-r_{2}}{\sqrt{\frac{1}{2T}(r_{1}+r_{2})}}=d'\text{ (21)}$.

Physiological data and numerical values for all plots can be found at osf.io/xhmus/.
